# Implementação da Pré-habilitação Cardíaca no Brasil: Construção de um Quadro Baseado em Evidências para Desfechos Cirúrgicos

**DOI:** 10.36660/abc.20250377

**Published:** 2025-12-22

**Authors:** Flavia Mazzoli-Rocha, Ricardo Stein, Carolin Steinmetz, Monika Sadlonova, Audrey Borghi-Silva, Thomas Schmidt

**Affiliations:** 1 Instituto Nacional de Infectologia Evandro Chagas Fundação Oswaldo Cruz Rio de Janeiro RJ Brasil Instituto Nacional de Infectologia Evandro Chagas, Fundação Oswaldo Cruz, Rio de Janeiro, RJ – Brasil; 2 Department of Preventive and Rehabilitative Sports and Performance Medicine Institute of Cardiology and Sports Medicine German Sport University Cologne Cologne Alemanha Department of Preventive and Rehabilitative Sports and Performance Medicine, Institute of Cardiology and Sports Medicine, German Sport University Cologne, Cologne – Alemanha; 3 Departamento de Clínica Médica Universidade Federal do Rio Grande do Sul Porto Alegre RS Brasil Departamento de Clínica Médica, Universidade Federal do Rio Grande do Sul, Porto Alegre, RS – Brasil; 4 Department of Geriatrics University Medical Center Goettingen Goettingen Alemanha Department of Geriatrics, University Medical Center Goettingen, Goettingen – Alemanha; 5 Department of Psychosomatic Medicine and Psychotherapy University Medical Center Goettingen Goettingen Alemanha Department of Psychosomatic Medicine and Psychotherapy, University Medical Center Goettingen, Goettingen – Alemanha; 6 Department of Cardiovascular and Thoracic Surgery University Medical Center Goettingen Goettingen Alemanha Department of Cardiovascular and Thoracic Surgery, University Medical Center Goettingen, Goettingen – Alemanha; 7 German Center for Cardiovascular Research Goettingen Alemanha German Center for Cardiovascular Research (DZHK), Goettingen – Alemanha; 8 Laboratório de Fisioterapia Cardiopulmonar Universidade Federal de São Carlos São Carlos SP Brasil Laboratório de Fisioterapia Cardiopulmonar, Universidade Federal de São Carlos, São Carlos, SP – Brasil; 9 Schüchtermann-Klinik Bad Rothenfelde Bad Rothenfelde Alemanha Schüchtermann-Klinik Bad Rothenfelde, Bad Rothenfelde – Alemanha

**Keywords:** Exercício Pré-Operatório, Cirurgia Torácica, Reabilitação Cardíaca

## Abstract

Pacientes submetidos à cirurgia cardíaca frequentemente enfrentam múltiplos desafios, incluindo comorbidades, fragilidade e fatores psicossociais, que complicam o processo de recuperação. Os programas de pré-habilitação multimodal têm surgido como uma estratégia promissora para abordar esses problemas, visando reduzir o tempo de internação hospitalar, mitigar as complicações pulmonares e melhorar a recuperação funcional.

Este Artigo de Revisão examina os componentes e os benefícios de um programa abrangente de pré-habilitação cardíaca, com foco na pré-habilitação baseada em exercícios. Adicionalmente, ressalta a importância da integração da pré-habilitação nas diretrizes clínicas brasileiras como uma oportunidade pré-operatória valiosa.

Evidências emergentes, embora de baixa a moderada qualidade, sugerem que a pré-habilitação baseada em exercício, como uma abordagem unimodal ou integrando um programa multimodal, pode melhorar significativamente a capacidade funcional e os resultados pós-operatórios em pacientes submetidos à cirurgia cardíaca eletiva. As diretrizes internacionais têm apoiado a pré-habilitação cardíaca como uma abordagem valiosa neste contexto. Globalmente, os ensaios de intervenção multicêntricos em andamento buscam refinar essa evidência, identificando a população ideal de pacientes e o programa de pré-habilitação cardíaca mais eficaz. Dada a crescente carga das doenças cardiovasculares no Brasil, há uma necessidade não satisfeita de pesquisa local para avaliar os benefícios da pré-habilitação em pacientes submetidos à cirurgia cardíaca.

## Graphical abstract

**Figura Central:**
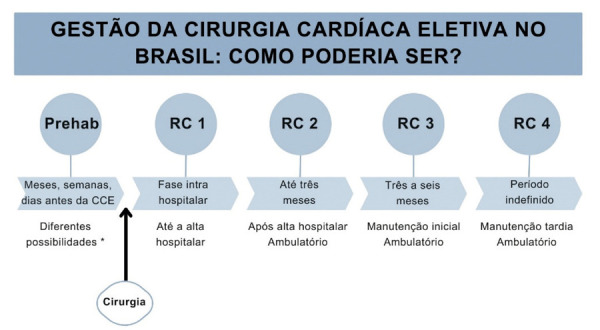
Implementação da Pré-habilitação Cardíaca no Brasil: Construção de um Quadro Baseado em Evidências para Desfechos Cirúrgicos A necessidade do reconhecimento das diretrizes nacionais. O reconhecimento da pré-habilitação no manejo de pacientes submetidos a cirurgias cardíacas eletivas nas diretrizes nacionais de cirurgia cardiovascular e reabilitação cardíaca poderia ser o primeiro passo. * A pré-habilitação pode ser realizada em diferentes cenários, incluindo internação hospitalar, ambulatório ou até mesmo por telepré-habilitação. Prehab: pré-habilitação; CCE: cirurgias cardíacas eletivas; RC 1: fase 1 da reabilitação cardíaca; RC 2: fase 2 da reabilitação cardíaca; RC 3: fase 3 da reabilitação cardíaca; RC 4: fase 4 da reabilitação cardíaca.

## Introdução

As doenças cardíacas isquêmicas e as doenças valvares cardíacas correspondem, respectivamente, à primeira e quinta principais causas de mortalidade e morbidade no mundo entre as doenças cardiovasculares.^1^ Para tratar essas condições, uma média global anual de 36,7 enxertos de revascularização do miocárdio e 30,8 cirurgias valvares ocorrem por 100.000 habitantes.^2^ Cirurgias envolvendo uma cavidade corporal maior, como a cirurgia cardíaca aberta, desencadeiam uma resposta neuroendócrina e inflamatória sistêmica, contribuindo para complicações pós-operatórias e aumento da mortalidade.^3^

Pacientes indicados para cirurgia cardíaca geralmente apresentam idade avançada, alta carga de comorbidades, fatores de risco dietéticos e riscos significativos de morbidade e mortalidade perioperatória, muitas vezes agravados pela fragilidade.^1 , 4^ A fragilidade afeta até 50% dos idosos submetidos à cirurgia cardíaca, estando fortemente ligada a desfechos adversos, como complicações pós-operatórias, altas taxas de mortalidade e hospitalização prolongada.^4 , 5^ Adicionalmente, a convalescença pode ser complicada por delírio, ansiedade e transtornos depressivos.^6^ Para mitigar esses riscos, a pré-habilitação surgiu como uma estratégia-chave para aumentar a resiliência física e otimizar pacientes frágeis para o estresse cirúrgico.^6^

Para reduzir as complicações pós-operatórias e melhorar a recuperação funcional em pacientes eleitos para cirurgia cardíaca, são necessárias mais pesquisas. Este Artigo de Revisão examina os componentes e os benefícios da pré-habilitação cardíaca abrangente, com foco em programas baseados em exercícios. Adicionalmente, ressalta a importância da integração da pré-habilitação nas diretrizes clínicas brasileiras como uma oportunidade pré-operatória valiosa.

### O Conceito de pré-habilitação

O conceito de pré-habilitação — também referido como reabilitação pré-operatória^5^ — decorre do reconhecimento de que a cirurgia bem-sucedida depende não só do procedimento em si, mas também da rápida recuperação física e psicológica do paciente.^7^ Um estudo inovador de 1981 com 29 pacientes cardíacos submetidos a cirurgia abdominal eletiva foi pioneiro em demonstrar que a pré-habilitação baseada em exercício (EBPrehab) poderia ajudar a avaliar e mitigar o risco cardíaco antes da cirurgia.^8^ Esta descoberta inicial estabeleceu as bases para a pré-habilitação evoluir para um elemento crítico dos cuidados cirúrgicos.

Em 2005, houve um avanço significativo nos cuidados perioperatórios com o desenvolvimento do protocolo *Enhanced Recovery After Surgery* (ERAS) para pacientes colorretais.^9^ Esse marco catalisou o crescente reconhecimento na literatura científica dos benefícios da pré-habilitação em várias especialidades cirúrgicas. Revisões sistemáticas e metanálises subsequentes demonstraram a eficácia da pré-habilitação multimodal em diversas populações de pacientes, incluindo ortopédica,^10^ abdominal,^11^ cardíaca,^12^ bariátrica^13^ e oncológica.^14^

Curiosamente, o desenvolvimento de programas de recuperação aprimorada para cirurgia cardíaca progrediu mais lentamente em comparação com os procedimentos não cardíacos. Esse atraso pode ser atribuído a vários desafios, incluindo a natureza de alto risco dos pacientes cardíacos e a considerável diversidade nas intervenções cirúrgicas cardíacas. Aproximadamente 15 anos após a introdução do protocolo ERAS, os princípios de recuperação aprimorada foram formalmente estabelecidos na cirurgia cardíaca,^15^ culminando na *Enhanced Recovery After Cardiac Surgery* (ERACS), uma das primeiras diretrizes abrangentes para o cuidado perioperatório nesta população.^16^

**Figura 1:**
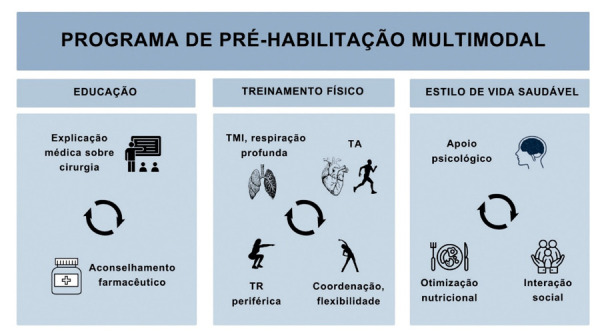
– Programa de pré-habilitação multimodal. Uma abordagem multidisciplinar de pré-habilitação que poderia ser incluída como intervenção antes das diferentes cirurgias, devendo ser apropriadamente individualizado.2,12,15 TMI: treinamento muscular inspiratório; TA: treinamento aeróbico; TR: treinamento de resistência.

Ao longo destes anos, a pré-habilitação evoluiu de uma abordagem unimodal (focada em uma única intervenção como exercício ou apoio psicológico) para uma estratégia multimodal. Como mostrado na Figura 1 , este modelo abrangente agora integra otimização médica, planejamento cirúrgico, suporte nutricional e psicológico, treinamento de exercícios e modificação do estilo de vida.^3 , 7 , 17 - 19^ Enquanto a evidência atual permanece limitada, as diretrizes do ERACS recomendam implementar tais programas de pré-habilitação multimodal para pacientes de cirurgia cardíaca eletiva.^5 , 16^ Como intervenção fundamental de pré-habilitação, o programa baseado em exercícios representa uma estratégia-chave para otimizar os resultados da cirurgia cardíaca.^12 , 20^

### Pré-habilitação baseada em exercício: uma estratégia-chave para melhorar os desfechos da cirurgia cardíaca?

**Figura 2:**
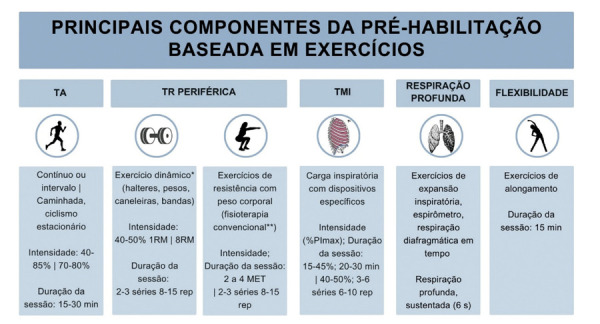
– Principais componentes da pré-habilitação baseada em exercício. * Principais grupos musculares dos membros superiores e inferiores. ** Sessões convencionais de fisioterapia. TA: treinamento aeróbico; TR: treinamento de resistência; 1RM: uma repetição máxima; 8RM: oito repetições máximas; rep: repetições; MET: equivalente metabólico da tarefa; TMI: treinamento muscular inspiratório; PImax: pressão inspiratória máxima. Baseado em 16 ensaios clínicos randomizados publicados anteriormente.21-36

A EBPrehab, intervenção fundamental da pré-habilitação cardíaca que envolve a aplicação terapêutica do exercício, tem sido cada vez mais incorporada nos cuidados cirúrgicos pré-operatórios.^20^ A Figura 2 apresenta uma visão geral dos componentes principais da EBPrehab com base em 16 ensaios clínicos randomizados^21 - 36^ que examinaram seus efeitos na cirurgia cardíaca, seja ela implementada como parte de programas de pré-habilitação multimodal ou unimodal. As intervenções EBPrehab diferiram em design, variando de abordagens de modalidade única a regimes combinados que incorporem pelo menos duas modalidades de treino, exercícios de expansão respiratória, ou exercícios de flexibilidade, conforme detalhado na Tabela 1 . Notavelmente, em 10 desses ECRs, a EBPrehab foi realizada por profissionais de fisioterapia ou implementada através de sessões estruturadas de fisioterapia.^23 - 28 , 30 , 31 , 35 , 36^

**Tabela 1:** – Pré-habilitação baseada em exercício em pacientes submetidos à cirurgia cardíaca

Artigo, Ano	País, Cenário	População, Tamanho da amostra ^*^	Modalidade Prehab: componentes	Detalhes da EBPrehab
Weiner, 1998^21^	Israel, Ambulatorial	CRM, N=42	Unimodal: EBPrehab	TMI : 2-4 semanas, 6 vezes por semana, 30 minutos a 15% PImax com aumento progressivo até 60% PImax
Arthur, 2000^22^	Canadá, Ambulatorial	CRM ^#^, N=123	Multimodal: Educação | EBPrehab	8 semanas, duas vezes por semana Flexibilidade : 20 minutos, alongamento TA : 30 minutos (ciclo estacionário, esteira, ergômetro do braço, escalador da escada) a 40-70% da capacidade funcional
Hulzebos, 2006^23^	Países Baixos, Ambulatorial e domiciliar	CRM ^#^, N=14	Unimodal: EBPrehab	TMI : pelo menos 2 semanas, 7 vezes por semana (1 na clínica e 6 em casa), 20 minutos a 30% PImax, supervisionado por um fisioterapeuta
Hulzebos, 2006^24^	Países Baixos, Ambulatorial e domiciliar	CRM ^#^, N=140	Unimodal: EBPrehab	TMI : pelo menos 2 semanas, 7 vezes por semana (1 na clínica e 6 em casa), 20 minutos a 30% PImax, supervisionado por um fisioterapeuta
Herdy, 2008^25^	Brasil, Hospitalar	CRM, N=29	Unimodal: EBPrehab	Pelo menos 5 dias, supervisionado por fisioterapeutas TR periférico : 2-4 METs com base na fase I da RC Respiração profunda : treinamento de espirômetro e RPPI
Ferreira, 2009^26^	Brasil, Domiciliar	CRM | CTV, N=15	Unimodal: EBPrehab	TMI : pelo menos 5 dias, 2 vezes ao dia, 5 séries, 10 repetições a 40% PImax (avaliada por fisioterapeutas)
Rosenfeldt, 2011^27^	Austrália, Ambulatorial	CRM ^#^, N=60	Unimodal: EBPrehab	TA : 2 semanas, duas vezes por semana, 40 minutos de cicloergometria, caminhada em esteira, ergometria de braço a 60% FCmax, supervisionado por fisioterapeutas
Savci, 2011^28^	Turquia, Hospitalar	CRM, N=22	Unimodal: EBPrehab	TMI : 5 dias, duas vezes ao dia, 30 minutos a 15% PImax com aumento progressivo até 45% PImax, supervisionado por fisioterapeutas
Sawatzky, 2014^29^	Canadá, Ambulatorial	CRM ^#^, N=8	Multimodal: Educação | EBPrehab | Estilo de vida saudável	TA : 4 semanas, duas vezes por semana, 60 minutos de caminhada a 85% VO_2_pico (alongamento e exercício leve com peso corporal e faixas foram realizados por apenas 2 participantes)
Sobrinho, 2014^30^	Brasil, Hospitalar	CRM, N=10	Unimodal: EBPrehab	Todos os dias até a cirurgia ^&^, uma sessão por dia, supervisionado por fisioterapeutas TMI : 3 séries, 10 repetições a 40% PImax Respiração profunda : inspiração profunda e sustentada, respiração diafragmática associado à mobilização dos membros superiores
Chen, 2019^31^	China, Hospitalar	CRM | CTV, N=98	Multimodal: Educação | EBPrehab	TMI : 5 dias, duas vezes por dia, 20 minutos (5 respirações diafragmáticas seguidas de 5-10 segundos de descanso) a 30% PImax, supervisionado por fisioterapeutas
Steinmetz, 2020^32^	Alemanha, Ambulatorial	CRM ^#^, N=81	Unimodal: EBPrehab	2 semanas, 3 vezes por semana TA : 2 sets, 10-25 minutos de cicloergometria a 70% VO_2_pico Respiração profunda : técnicas respiratórias
Argunova, 2022^33^	Rússia, Ambulatorial	CRM ^#^, N=43	Unimodal: EBPrehab	TA : 5-10 dias, 30 minutos a 80% VO_2_pico
Akowuah, 2023^34^	Reino Unido, Ambulatorial e domiciliary	CRM ^#^, N=91	Multimodal: Educação | EBPrehab	4 semanas, 7 vezes por semana (1 na clínica e 6 em casa), 45-60 minutos TR periférico : programa de exercícios TMI : duas vezes por dia, em alta intensidade
López-Hernández, 2024^35^	Espanha, Ambulatorial	CTV, N=34	Multimodal: EBPrehab | Estilo de vida saudável	4-6 semanas, duas vezes por semana, 60 minutos por sessão, supervisionado por fisioterapeutas TA : 5 períodos de 2 minutos de cicloergometria, 70% a 90-100% PWR TR periférico : 2-3 séries, 8-12 repetições, 8RM (peitoral, grande dorsal e quadríceps) Respiração profunda : duas vezes por dia, 1-2 séries, 10-15 respirações (expansões torácicas, respirações diafragmáticas e inspirações profundas)
Sahar, 2024^36^	Paquistão, Ambulatorial	CRM ^#^, N=37	Unimodal: EBPrehab	TR periférico : 1-3 vezes ao dia, 10-15 repetições por grupos musculares principais (superior e inferior), com caneleiras/halteres a 40-50% 1RM, supervisionado por fisioterapeutas

CRM: cirurgia de revascularização do miocárdio; CTV: cirurgia de troca valvar; EBPrehab: exercise-based prehabilitation; FCmax: frequência cardíaca máxima; PImax: pressão inspiratória máxima; Prehab: pré-habilitação; PWR: carga de trabalho máxima; RC: reabilitação cardíaca; RPPI: respiração com pressão positiva intermitente; TA: treinamento aeróbico;

TMI: treinamento muscular inspiratório; TR: treinamento de resistência; VO_2_pico: consumo máximo de oxigênio; 1RM: uma repetição máxima; 8RM: oito repetições máximas. *Participantes incluídos no grupo de intervenção Pré-habilitação. ^#^Especificado como CRM eletiva. & Duração da EBPrehab não especificada no artigo.

As evidências atuais mostram uma variabilidade considerável na prescrição de exercícios para pacientes cirúrgicos cardíacos, sem um consenso estabelecido sobre a modalidade ideal ou parâmetros de dosagem. Os programas existentes demonstram grandes variações na duração (de 5 dias a 16 semanas), frequência (2 a 7 sessões por semana), duração da sessão (20 a 90 minutos) e intensidade (baixa-moderada a alta).^20^ Apesar da alta heterogeneidade nos protocolos de intervenção, diferentes revisões sistemáticas e metanálises consistentemente apoiam a EBPrehab para melhorar a capacidade funcional pós-operatória,^12 , 20 , 37 , 38^ reduzir as complicações pós-operatórias^12 , 20 , 37 , 39^ e diminuir o tempo de internação hospitalar^12 , 20 , 37 - 39^ em pacientes submetidos à cirurgia cardíaca. As evidências sugerem que esses benefícios são reprodutíveis em diferentes programas, ressaltando a robustez da EBPrehab nos cuidados cirúrgicos cardíacos eletivos.

Uma recente declaração de consenso europeu propõe que a prescrição da EBPrehab deve incorporar treino físico e treino dos músculos respiratórios, individualizado de acordo com as necessidades específicas do paciente e fatores de risco — particularmente para populações frágeis.^6^ Notavelmente, o treinamento muscular inspiratório ganhou reconhecimento como uma intervenção eficaz, econômica e bem tolerada. Esta modalidade funciona aplicando resistência durante a inspiração para aumentar força e resistência muscular inspiratória.^40^ Quando implementado como uma intervenção independente, o treinamento muscular inspiratório tem demonstrado impacto significativo na redução de complicações pulmonares pós-operatórias (incluindo atelectasia e pneumonia) e tempo de recuperação.^41 - 44^

**Figura 3:**
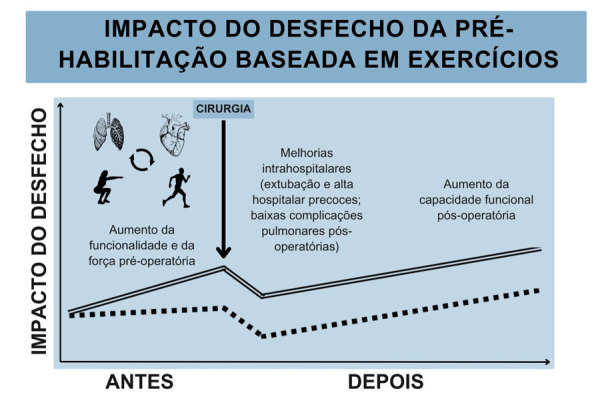
– Impacto do desfecho da pré-habilitação baseada em exercício na cirurgia cardíaca eletiva. Ilustração sobre o impacto do desfecho antes e após a cirurgia cardíaca, na presença (linha dupla) ou ausência (linha pontilhada) da pré-habilitação baseada em exercício. Baseado em 15.

Evidências existentes, embora de qualidade baixa a moderada, indicam que a EBPrehab melhora a capacidade funcional e facilita a recuperação pós-operatória em pacientes de cirurgia cardíaca eletiva ( Figura 3 ).^12 , 37 - 39 , 41^ Uma abordagem combinada de exercícios que incorpora exercícios de expansão respiratória, treinamento muscular inspiratório, treinamento de resistência periférica e condicionamento físico geral,^41 , 42 , 44^ parece particularmente benéfica, ressaltando o valor da implementação precoce e abrangente da EBPrehab.

Atualmente, não há evidências suficientes para recomendar um protocolo de intervenção padronizado ou uma combinação ótima dos componentes da EBPrehab. Uma abordagem individualizada para EBPrehab é, portanto, justificada e muitas vezes necessária. Por exemplo, pode nem sempre ser viável ou clinicamente necessário incorporar todos os componentes da EBPrehab em uma única sessão, especialmente para pacientes frágeis com grande descondicionamento muscular, pois podem não ter a capacidade de tolerar tais volumes de treinamento extensos.

Apesar da necessidade de pesquisas adicionais de alta qualidade, as atuais diretrizes internacionais endossam a pré-habilitação como uma estratégia valiosa para otimização pré-operatória em cirurgia cardíaca eletiva.^5 , 6 , 16^ Visando os fatores de risco prevalentes nesta população^4 , 6 , 45^ — particularmente fragilidade nos idosos — a EBPrehab representa uma intervenção clinicamente significativa para melhorar os resultados cirúrgicos. Alcançar esses resultados requer colaboração multiprofissional entre todos os profissionais de saúde, com cada disciplina contribuindo com sua experiência especializada. Os médicos devem assumir a responsabilidade pela avaliação de risco e estabelecer as restrições clínicas necessárias. Para intervenções específicas como aconselhamento nutricional ou cessação do tabagismo, o envolvimento de outros especialistas — incluindo nutricionistas e psicólogos — é essencial.

### Pré-habilitação cardíaca baseada em exercício na prática clínica no Brasil

A EBPrehab — também conhecida como fisioterapia pré-operatória^24 , 26 - 28 , 30 , 31 , 35 , 36 , 41^ — está se tornando cada vez mais comum no Brasil. O Conselho Federal de Fisioterapia e Terapia Ocupacional (COFFITO) reconhece quinze especialidades em fisioterapia, duas das quais voltadas para os cuidados cardíacos: fisioterapia cardiovascular e fisioterapia intensiva.^46 - 48^ Os fisioterapeutas cardiovasculares e de terapia intensiva podem contribuir para o componente de EBPrehab nos programas de pré-habilitação cardíaca em três cenários clínicos distintos.

Primeiro, os pacientes de alto risco que necessitam de preparação pré-operatória intensiva recebem cuidados multimodais nas unidades de terapia intensiva. Segundo, os centros convencionais de reabilitação cardíaca servem como locais estabelecidos para a entrega do programa multimodal. Nestes dois cenários, a abordagem centralizada multimodal é realizada por meio de intervenções médicas, fisioterapêuticas, nutricionais e psicológicas coordenadas.^3 , 7^ Terceiro, pacientes estáveis aguardando cirurgia eletiva podem ser submetidos a EBPrehab em clínicas de fisioterapia cardíaca após avaliação de risco e referência médica. Neste caso, o programa multimodal pode ser entregue através de uma abordagem descentralizada.^47^ Em todos estes cenários, os fisioterapeutas, de forma autônoma, avaliam capacidade funcional e funcionalidade, estabelecem perfis diagnósticos e projetam programas individualizados de EBPrehab com base nos fatores de risco de cada paciente e no tempo restante antes da cirurgia.^47 , 48^

No entanto, essas intervenções de exercício até agora são geralmente baseadas em prescrições clínicas individualizadas, em vez de protocolos padronizados, sublinhando uma desconexão entre a prática clínica e a pesquisa baseada em evidências. Poucos estudos brasileiros investigaram os benefícios da EBPrehab em programas de fisioterapia pré-operatória para pacientes submetidos à cirurgia de revascularização miocárdica. Um estudo realizado em Santa Catarina^25^ demonstrou que a EBPrehab (consistindo de pelo menos cinco dias de exercícios de resistência ao peso corporal e exercícios respiratórios) combinado com a reabilitação cardíaca Fase I reduziu significativamente as permanências hospitalares, tempo para extubação e a incidência de complicações pós-operatórias — incluindo pneumonia, derrame pleural e atelectasia — em comparação com a reabilitação cardíaca Fase I sozinha. Adicionalmente, dois estudos de São Paulo revelaram que um programa de EBPrehab com treinamento muscular inspiratório pode reduzir o tempo de recuperação pós-operatória^30^ e melhorar a função pulmonar.^26^

Apesar desses resultados promissores, a reabilitação cardíaca pré-operatória (ou “pré-habilitação cardíaca”) permanece subutilizada no Brasil.

### Pré-habilitação cardíaca no Brasil: Uma necessidade emergente

Apesar de mais de quatro décadas de pesquisa apoiando os benefícios da pré-habilitação,^2^ — incluindo sua recomendação em diretrizes internacionais para pacientes submetidos a cirurgia cardíaca eletiva^5 , 6 , 16^ — a pré-habilitação ainda não foi reconhecida como uma oportunidade valiosa de intervenção nas diretrizes brasileiras.

A Diretriz Sul-Americana para Prevenção e Reabilitação Cardiovascular (2014)^49^ recomenda reabilitação cardíaca pós-operatória para procedimentos como revascularização miocárdica e transplante cardíaco, mas não faz menção a intervenções pré-operatórias como pré-habilitação. Da mesma forma, o mais recente documento abrangente do Brasil sobre o tema, a Diretriz Brasileira para Reabilitação Cardiovascular (2020),^50^ omite a pré-habilitação como prática recomendada.

Esta situação se estende a outras diretrizes importantes:

- A 3ª Diretriz Brasileira de Transplante Cardíaco (2018)51 se concentra exclusivamente na avaliação médica pré-operatória sem referenciar programas de pré-habilitação.
- A Diretriz atualizada para Avaliação Cardiovascular Perioperatória (2024) da Sociedade Brasileira de Cardiologia52 enfatiza a avaliação de risco, mas não inclui referências de pré-habilitação — mesmo para pacientes de alto risco. Notavelmente, sua única menção de reabilitação física se aplica exclusivamente a pacientes frágeis submetidos a procedimentos não cardíacos.

Esta exclusão persistente nas orientações nacionais representa uma oportunidade perdida. Dada a crescente evidência que demonstra a eficácia da pré-habilitação na melhoria dos resultados cirúrgicos, o Brasil pode se beneficiar significativamente ao alinhar sua prática clínica com os padrões internacionais através da incorporação formal de protocolos de pré-habilitação.

### Desafios antecipados e soluções propostas para implementação na rotina clínica

Inevitavelmente, a implementação de programas de pré-habilitação cardíaca no Brasil enfrentará desafios. Embora ainda sejam desconhecidas as barreiras específicas à participação na pré-habilitação cardíaca, a literatura existente destaca desafios sistêmicos na prestação de serviços de reabilitação cardíaca.

Os serviços de reabilitação cardíaca nos países de baixa e média renda são insuficientes para enfrentar a crescente carga das doenças cardiovasculares, com baixa utilização mesmo quando disponíveis. Embora existam barreiras em todos os cenários, os países de renda baixa e média enfrentam maiores desafios devido à escassez de recursos, sistemas de saúde fracos e questões de acessibilidade.^53^ As principais barreiras de implementação incluem baixa motivação do paciente, desafios ambientais e dietéticos, lacunas de infraestrutura e disparidades na alfabetização em saúde. A cobertura limitada do seguro restringe ainda mais o acesso e o potencial de reabilitação a longo prazo.^54 , 55^ Para pacientes idosos com fragilidade, as barreiras da pré-habilitação incluem limitações de transporte, falta de apoio social e sobrecarga de informações.^56 , 57^

Dada a infraestrutura limitada de reabilitação cardíaca no Brasil para programas centralizados de pré-habilitação multimodal, estratégias adaptativas de implementação são essenciais. Os desafios associados aos programas baseados em centros poderiam ser mitigados através de modelos descentralizados e/ou teleprehabilitação, que não apenas abordem essas barreiras, mas que também podem melhorar a relação custo-benefício.^58 , 59^ Pesquisas futuras devem explorar modelos adaptados localmente que alavanquem a colaboração interprofissional. Por questões de segurança, pacientes de risco moderado a alto devem ser encaminhados para programas baseados em hospitais ou centros, enquanto os pacientes de baixo risco que aguardam uma cirurgia eletiva podem receber cuidados multidisciplinares coordenados e descentralizados em ambientes comunitários ou através da tele-reabilitação. Tais programas devem integrar componentes dos domínios médico, fisioterapêutico, nutricional e psicológico, dependendo das necessidades individuais, para garantir um apoio abrangente.

### Perspectivas de futuro: Avanço da pré-habilitação cardíaca no Brasil

Enquanto as lacunas de evidência persistem e não existe consenso sobre os programas ideais de pré-habilitação, as sociedades médicas internacionais reconhecem cada vez mais a necessidade urgente de estudos de alta qualidade neste campo.^5 , 6^

Nos últimos anos temos visto inúmeras iniciativas multinacionais investigando estratégias eficazes de pré-habilitação para pacientes cardíacos. Programas notáveis incluem abordagens multimodais (ERAS,^5^ PREHAB,^60^ PREQUEL,^61^ PRECOVERY,^62^ PrEPS TRIAL,^63^ Heart-ROCQ^64^) e pesquisa em telepré-habilitação,^65 , 59^ que mostram a promessa em melhorar resultados para pacientes cirúrgicos cardíacos, incluindo aqueles submetidos ao transplante cardíaco.^66^

Para estabelecer uma estrutura de pré-habilitação baseada em evidências, adaptada às realidades da população e da saúde no Brasil, as seguintes prioridades devem ser consideradas:

### Principais recomendações para o Brasil

1. **Integração de Diretrizes**
  - Reconhecimento formal da pré-habilitação multimodal nas diretrizes nacionais de cirurgia cardiovascular e reabilitação cardíaca como uma oportunidade valiosa de intervenção para a cirurgia cardíaca eletiva.
2. **Pesquisa Específica da População**
  - Investigação dos fatores epidemiológicos e culturais únicos do Brasil para garantir que os programas de pré-habilitação lidem com as disparidades locais em saúde e restrições de recursos.
3. **Implementação Multidisciplinar**
  - Estabelecimento de equipes especializadas em pré-habilitação (cardiologistas, fisioterapeutas, nutricionistas, psicólogos) para projetar programas adaptados ao contexto e alinhados à infraestrutura de saúde do Brasil.
4. **Programas-piloto em Centros Acadêmicos**
  - Implantação de iniciativas de pré-habilitação em hospitais universitários e centros de reabilitação cardíaca para sinergizar o cuidado clínico com a pesquisa enquanto se testa a viabilidade.
5. **Expansão para Procedimentos Cardíacos Fechados**
  - Avaliação rigorosa do papel da pré-habilitação em intervenções coronarianas percutâneas, implantação de válvula aórtica transcateter e outros procedimentos cardíacos minimamente invasivos.
6. **Otimização da Modalidade do Exercício**
  - Estudos comparativos para determinar a segurança e a eficácia de vários componentes de treinamento (aeróbio, resistência e/ou inspiratório) dentro de estruturas de pré-habilitação multimodal.

## Conclusão: Um chamado à ação para a pré-habilitação cardíaca no Brasil

Apesar da evidência crescente de apoio à reabilitação pré-operatória para cirurgia cardíaca, o sistema de saúde do Brasil não implementou totalmente essa abordagem. Enquanto as diretrizes internacionais reconhecem cada vez mais a pré-habilitação como um componente valioso da preparação cirúrgica,^5 , 6 , 16^ sua ausência nas orientações cardiovasculares brasileiras^49 - 52^ representa uma lacuna crítica nos padrões de atendimento ao paciente. A comunidade médica brasileira, junto com os demais profissionais de saúde, se encontra em um momento crucial:

1. **Incorporação formal** da pré-habilitação nas diretrizes nacionais de cuidados cardíacos.
2. **Desenvolvimento de protocolos baseados em evidências** adaptados às necessidades da população local e à infraestrutura de saúde.
3. **Estabelecimento de programas multidisciplinares** em centros acadêmicos de saúde.

Ao abraçar esses avanços, o Brasil tem potencial para melhorar significativamente os resultados da cirurgia cardíaca. O sucesso dos programas multimodais internacionais^60 - 64^ e modelos emergentes de telepré-habilitação^59 , 65 , 66^ fornece um roteiro e uma justificativa para esta evolução. A investigação futura deve centrar-se particularmente em:

- padronizar as medidas de desfecho para as populações brasileiras,
- avaliar a relação custo-efetividade nos sistemas de saúde públicos e privados e
- explorar as aplicações em procedimentos cardíacos minimamente invasivos,
- para melhorar o conhecimento existente e otimizar a situação de saúde.
