# Supplementary material for: Implementação da Pré-habilitação Cardíaca no Brasil: Construção de um Quadro Baseado em Evidências para Desfechos Cirúrgicos
Source: Arq Bras Cardiol. 2025 Dec 22;122(10):e20250377. [Article in Portuguese] doi: 10.36660/abc.20250377 (PMC12978167; doi:10.36660/abc.20250377)
Supplement: Translation

# Translation: Implementing Cardiac Prehabilitation in Brazil: Building an Evidence-based Framework for Surgical Outcomes

**DOI:** 10.36660/abc.20250377i

**Authors:** Flavia Mazzoli-Rocha, Ricardo Stein, Steinmetz Carolin, Monika Sadlonova, Audrey Borghi-Silva, Thomas Schmidt

**Affiliations:** 1 Brazil Instituto Nacional de Infectologia Evandro Chagas, Fundação Oswaldo Cruz, Rio de Janeiro, RJ – Brazil; 2 Germany Department of Preventive and Rehabilitative Sports and Performance Medicine, Institute of Cardiology and Sports Medicine, German Sport University Cologne, Cologne – Germany; 3 Brazil Departamento de Clínica Médica, Universidade Federal do Rio Grande do Sul, Porto Alegre, RS – Brazil; 4 Germany Department of Geriatrics, University Medical Center Goettingen, Goettingen – Germany; 5 Germany Department of Psychosomatic Medicine and Psychotherapy, University Medical Center Goettingen, Goettingen – Germany; 6 Germany Department of Cardiovascular and Thoracic Surgery, University Medical Center Goettingen, Goettingen – Germany; 7 Germany German Center for Cardiovascular Research (DZHK), Goettingen – Germany; 8 Brazil Laboratório de Fisioterapia Cardiopulmonar, Universidade Federal de São Carlos, São Carlos, SP – Brazil; 9 Germany Schüchtermann-Klinik Bad Rothenfelde, Bad Rothenfelde – Germany

**Keywords:** Preoperative Exercise, Thoracic Surgery, Cardiac Rehabilitation

## Abstract

Patients undergoing cardiac surgery often face multiple challenges, including comorbidities, frailty, and psychosocial factors, which complicate recovery. Multimodal prehabilitation programs have emerged as a promising strategy to address these issues, aiming to reduce the length of hospital stay, mitigate pulmonary complications, and enhance functional recovery.

This Review Article examines the components and benefits of a comprehensive cardiac prehabilitation program, with a focus on exercise-based prehabilitation. Additionally, it underscores the importance of integrating prehabilitation into Brazilian clinical guidelines as a valuable preoperative opportunity.

Emerging evidence, albeit of low to moderate quality, suggests that exercise-based prehabilitation, as a unimodal approach or integrating a multimodal program, can significantly improve functional capacity and postoperative outcomes in patients undergoing elective cardiac surgery. International guidelines have already supported cardiac prehabilitation as a valuable approach in this context. Globally, ongoing multicenter intervention trials seek to refine this evidence, pinpointing the ideal patient population and the most effective cardiac prehabilitation program. Given the growing burden of cardiovascular disease in Brazil, there is an unmet need for local research to assess the benefits of prehabilitation in patients undergoing cardiac surgery.

## Introduction

Ischemic heart diseases and heart valve diseases are the first and fifth leading causes of mortality and morbidity worldwide, respectively, among cardiovascular diseases.^1^To treat these conditions, an annual global average of 36.7 coronary artery bypass grafts and 30.8 valvular surgeries is performed per 100,000 population.^2^Surgeries involving a major body cavity, as open-heart surgery, trigger a systemic neuroendocrine and inflammatory response, contributing to postoperative complications and increased mortality.^3^

Cardiac surgery patients generally present with advanced age, high burden of comorbidities, dietary risk factors, and significant perioperative morbidity and mortality risks, often compounded by frailty.^1 , 4^Frailty affects up to 50% of older adults undergoing cardiac surgery and is strongly linked to adverse outcomes, including postoperative complications, high mortality rates, and prolonged hospitalization.^4 , 5^Furthermore, convalescence may be complicated by delirium, anxiety, and depressive disorders.^6^To mitigate these risks, prehabilitation has emerged as a key strategy to enhance physical resilience and optimize frail patients for surgical stress.^6^

To reduce postoperative complications and enhance functional recovery in cardiac surgery patients, further research is needed. This Review Article examines the components and benefits of comprehensive cardiac prehabilitation, with a focus on exercise-based programs. Additionally, it underscores the importance of integrating prehabilitation into Brazilian clinical guidelines as a valuable preoperative opportunity.

### The prehabilitation concept

The concept of prehabilitation — also mentioned as preoperative rehabilitation^5^— stems from the recognition that successful surgery depends not only on the procedure itself but also on the patient’s rapid physical and psychological recovery.^7^A groundbreaking 1981 study of 29 cardiac patients undergoing elective abdominal surgery was among the first to demonstrate that exercise-based prehabilitation (EBPrehab) could help assess and mitigate cardiac risk before surgery.^8^This early finding laid the groundwork for prehabilitation to evolve into a critical element of surgical care.

In 2005, a significant advancement in perioperative care occurred with the development of the Enhanced Recovery After Surgery (ERAS) protocol for colorectal patients.^9^This milestone catalyzed growing recognition in the scientific literature of prehabilitation’s benefits across multiple surgical specialties. Subsequent systematic reviews and meta-analyses have demonstrated the effectiveness of multimodal prehabilitation in diverse patient populations, including orthopedic,^10^abdominal,^11^cardiac,^12^bariatric,^13^and cancer.^14^

Interestingly, the development of enhanced recovery programs for cardiac surgery progressed more slowly compared to non-cardiac procedures. This delay can be attributed to several challenges, including the high-risk nature of cardiac patients and the considerable diversity in cardiac surgical interventions. Nearly 15 years after the introduction of the ERAS protocol, enhanced recovery principles were formally established in cardiac surgery,^15^culminating in the Enhanced Recovery After Cardiac Surgery (ERACS) Society’s first comprehensive guidelines for perioperative care in this population.^16^

Through these years, prehabilitation evolved from a unimodal approach (focused on a single intervention like exercise or psychological support) to a multimodal strategy. As shown in Figure 1 , this comprehensive model now integrates medical optimization, surgical planning, nutritional and psychological support, exercise training, and lifestyle modification.^3 , 7 , 17 - 19^While current evidence remains limited, the ERACS guidelines recommend implementing such multimodal prehabilitation programs for elective cardiac surgery patients.^5 , 16^As a fundamental intervention of prehabilitation, the exercise-based program represents a key strategy for optimizing outcomes in cardiac surgery.^12 , 20^

**Figure 1:** – Multimodal prehabilitation program. A multidisciplinary prehabilitation approach that could be included as an intervention before different surgeries, and should be individualized accordingly.2,12,15 IMT: inspiratory muscle training; AT: aerobic training; RT: resistance training.

### Exercise-based prehabilitation: a key strategy for improving cardiac surgical outcomes?

EBPrehab, a fundamental intervention of cardiac prehabilitation involving the therapeutic application of exercise, has been increasingly incorporated into preoperative surgical care.^20^ Figure 2 provides an overview of EBPrehab core components based on 16 randomized controlled trials^21 - 36^examining its effects in cardiac surgery, whether implemented as part of multimodal or unimodal prehabilitation programs. The EBPrehab interventions varied in design, ranging from single-component approaches to combined regimens incorporating at least two training modalities, breathing expansion exercises, or flexibility exercises, as detailed in Table 1 . Notably, in 10 of these RCTs, EBPrehab was either conducted by physiotherapy professionals or implemented through structured physiotherapy sessions.^23 - 28 , 30 , 31 , 35 , 36^

**Figure 2:** – Core components of exercise-based prehabilitation. * Major muscle groups of the upper and lower limbs. ** Conventional sessions of physiotherapy. AT: aerobic training; RT: resistance training; 1RM: one-repetition maximum; 8RM: eight-repetition maximum; rep: repetitions; MET: metabolic equivalent of task; IMT: inspiratory muscle training; MIP: maximum inspiratory pressure. Based on 16 previously published randomized controlled trials. 21-36

**Table 1:** – Exercise-based prehabilitation in patients undergoing cardiac surgery

Study, Year	Country, Setting	Population, Sample size^*^	Prehab modality: components	Details of EBPrehab
Weiner, 1998^21^	Israel, Outpatient clinic	CABG, N=42	Unimodal: EBPrehab	IMT : 2-4 weeks, 6 times weekly, 30 minutes at 15% MIP with progressive increase up to 60% MIP
Arthur, 2000^22^	Canada, Outpatient clinic	CABG^#^, N=123	Multimodal: Education | EBPrehab	8 weeks, twice weekly Flexibility : 20 minutes, stretching AT : 30 minutes (stationary cycle, treadmill, arm ergometer, stair climber) at 40-70% of functional capacity
Hulzebos, 2006^23^	The Netherlands, Outpatient clinic and Home	CABG^#^, N=14	Unimodal: EBPrehab	IMT : at least 2 weeks, 7 times weekly (1 at clinic and 6 at home), 20 minutes at 30% MIP, supervised by a physiotherapist
Hulzebos, 2006^24^	The Netherlands, Outpatient clinic and Home	CABG^#^, N=140	Unimodal: EBPrehab	IMT : at least 2 weeks, 7 times weekly (1 at clinic and 6 at home), 20 minutes at 30% MIP, supervised by physiotherapists
Herdy, 2008^25^	Brazil, Hospital	CABG, N=29	Unimodal: EBPrehab	At least 5 days, supervised by physiotherapists Peripheral RT : 2-4 METs based on CR phase I Breathing expansion : spirometer training and IPPB
Ferreira, 2009^26^	Brazil, Home	CABG | VRS, N=15	Unimodal: EBPrehab	IMT : at least 5 days, 2 times daily, 5 sets, 10 repetitions at 40% MIP (assessed by physiotherapists)
Rosenfeldt, 2011^27^	Australia, Outpatient clinic	CABG^#^, N=60	Unimodal: EBPrehab	AT : 2 weeks, twice weekly, 40 minutes of cycle ergometry, treadmill walking, arm ergometry at 60% HRmax, supervised by physiotherapists
Savci, 2011^28^	Turkey, Hospital	CABG, N=22	Unimodal: EBPrehab	IMT : 5 days, twice daily, 30 minutes at 15% MIP with progressive increase up to 45% MIP, supervised by physiotherapists
Sawatzky, 2014^29^	Canada, Outpatient clinic	CABG^#^, N=8	Multimodal: Education | EBPrehab | Healthy lifestyle	AT : 4 weeks, twice weekly, 60 minutes walking at 85% VO_2_peak (stretching and light exercise with body weight and bands were performed by only 2 participants)
Sobrinho, 2014^30^	Brasil, Hospital	CABG, N=10	Unimodal: EBPrehab	Daily until surgery &, once a day, supervised by physiotherapists IMT : 3 sets, 10 repetitions at 40% MIP Breathing expansion : deep and sustained inspiration, diaphragmatic breathing together with upper limbs mobilization
Chen, 2019^31^	China, Hospital	CABG | VRS, N=98	Multimodal: Education | EBPrehab	IMT : 5 days, twice daily, 20 minutes (5 diaphragmatic breaths followed by 5-10 seconds rest) at 30% MIP, supervised by physiotherapists
Steinmetz, 2020^32^	Germany, Outpatient clinic	CABG^#^, N=81	Unimodal: EBPrehab	2 weeks, 3 times weekly AT: 2 sets, 10-25 minutes cycle ergometer at 70% VO_2_peak Breathing expansion : breathing techniques
Argunova, 2022^33^	Russia, Outpatient clinic	CABG^#^, N=43	Unimodal: EBPrehab	AT : 5-10 days, 30 minutes at 80% VO_2_peak
Akowuah, 2023^34^	United Kingdom, Outpatient clinic and home	CABG^#^, N=91	Multimodal: Education | EBPrehab	4 weeks, 7 times weekly (1 at clinic and 6 at home), 45-60 minutes Peripheral RT : exercise programme IMT : twice daily, at high intensity
López-Hernández, 2024^35^	Spain, Outpatient clinic	VRS, N=34	Multimodal: EBPrehab | Healthy lifestyle	4-6 weeks, twice weekly, 60 minutes per session, supervised by physiotherapists AT : 5 bouts of 2 minutes, stationary bike at 70% to 90-100% PWR Peripheral RT: 2-3 sets, 8-12 repetitions, at 8RM (pectorals, latissimus dorsi, and quadriceps) Breathing expansion : twice daily, 1-2 sets, 10-15 breaths (chest expansions, diaphragmatic breaths, and deep inspirations)
Sahar, 2024^36^	Pakistan, Outpatient clinic	CABG^#^, N=37	Unimodal: EBPrehab	Peripheral RT : 1-3 times daily, 10-15 repetitions per major muscle groups (upper and lower), with cuff weights/dumbbells at 40-50% 1RM, supervised by physiotherapists

AT: aerobic training; CABG: coronary artery bypass graft; CR: cardiac rehabilitation; EBPrehab: exercise-based prehabilitation; IMT: inspiratory muscle training; IPPB: intermittent positive pressure breathing; HRmax: maximum heart rate; MIP: maximum inspiratory pressure; Prehab: prehabilitation; PWR: peak workload; RT: resistance training; VO_2_peak: peak oxygen consumption; VRS: valve replacement surgery; 1RM: one-repetition maximum; 8RM: eight-repetition maximum. *Participants included in the Prehabilitation intervention group. # Specified as elective CABG. & EBPrehab duration not specified in the study.

Current evidence shows considerable variability in exercise prescription for cardiac surgery patients, with no established consensus on optimal modality or dosage parameters. Existing programs demonstrate wide variations in duration (from 5 days to 16 weeks), frequency (2 to 7 sessions per week), session length (20 to 90 minutes), and intensity (low-moderate to high).^20^Despite the high heterogeneity in intervention protocols, different systematic reviews and meta-analyses consistently support EBPrehab for enhancing postoperative functional capacity,^12 , 20 , 37 , 38^reducing postoperative complications,^12 , 20 , 37 , 39^and decreasing length of hospital stay^12 , 20 , 37 - 39^in patients undergoing cardiac surgery. The evidence suggests these benefits are reproducible across different program designs, underscoring the robustness of EBPrehab in elective cardiac surgical care.

A recent European consensus statement proposes that the prescription of EBPrehab should incorporate physical exercise training and respiratory muscles training, individualized according to patient-specific needs and risk factors — particularly for frail populations.^6^Notably, inspiratory muscle training has gained recognition as an effective, cost-efficient, and well-tolerated intervention. This modality works by applying resistance during inspiration to enhance inspiratory muscle strength and endurance.^40^When implemented as a standalone intervention, inspiratory muscle training has demonstrated a significant impact in reducing postoperative pulmonary complications (including atelectasis and pneumonia) and recovery time.^41 - 44^

Existing evidence, albeit of low to moderate quality, indicates that EBPrehab improves functional capacity and facilitates postoperative recovery in elective cardiac surgery patients ( Figure 3 ).^12 , 37 - 39 , 41^A combined exercise approach incorporating breathing expansion exercises, inspiratory muscle training, peripheral resistance training, and general physical conditioning,^41 , 42 , 44^appears particularly beneficial, underscoring the value of early and comprehensive EBPrehab implementation.

**Figure 3:** – Outcome impact of an exercise-based prehabilitation in elective cardiac surgery. Illustration about the outcome impact before and after cardiac surgery, in the presence (double line) or absence (dotted line) of an exercise-based prehabilitation. Based on 15.

Currently, there is insufficient evidence to recommend a standardized intervention protocol or optimal combination of EBPrehab components. An individualized approach to EBPrehab is therefore warranted and often needed. For instance, it may not always be feasible or clinically necessary to incorporate all EBPrehab components within a single session, particularly for frail patients with marked muscular deconditioning, as they may lack the capacity to tolerate such extensive training volumes.

Although additional high-quality research is warranted, current international guidelines endorse prehabilitation as a valuable strategy for preoperative optimization in elective cardiac surgery.^5 , 6 , 16^By targeting prevalent risk factors in this population^4 , 6 , 45^— particularly frailty in the elderly — EBPrehab represents a clinically significant intervention for enhancing surgical outcomes. Achieving these outcomes requires multiprofessional collaboration among all healthcare providers, with each discipline contributing to their specialized expertise. Physicians should assume responsibility for risk assessment and establish necessary clinical restrictions. For specific interventions such as nutritional counseling or smoking cessation, the involvement of other specialists — including nutritionists and psychologists — is essential.

### Exercise-based cardiac prehabilitation in clinical practice in Brazil

EBPrehab — also known as preoperative physiotherapy^24 , 26 - 28 , 30 , 31 , 35 , 36 , 41^— is becoming increasingly common in Brazil. The Brazilian Federal Council of Physiotherapy and Occupational Therapy (COFFITO) recognizes fifteen specialties in physiotherapy, two of which focus on cardiac care: cardiovascular physiotherapy and intensive care physiotherapy.^46 - 48^Cardiovascular and intensive care physiotherapists can contribute to the EBPrehab component of cardiac prehabilitation programs across three distinct clinical scenarios.

First, high-risk patients necessitating intensive preoperative preparation receive multimodal care in intensive care units. Second, conventional cardiac rehabilitation centers serve as established sites for multimodal program delivery. In these two scenarios, the multimodal centralized approach is delivered through coordinated medical, physiotherapeutic, nutritional, and psychological interventions.^3 , 7^Third, stable patients awaiting elective surgery may undergo EBPrehab in cardiac physiotherapy clinics following physician risk assessment and referral. In this case, the multimodal program can be delivered through a decentralized approach.^47^In all scenarios, physiotherapists autonomously assess functional capacity and functionality, establish diagnostic profiles, and design individualized EBPrehab programs based on each patient’s risk factors and the time remaining before surgery.^47 , 48^

However, these exercise interventions are so far usually based on individualized clinical prescriptions rather than standardized protocols, underscoring a disconnect between clinical practice and evidence-based research. Few Brazilian studies have investigated the benefits of EBPrehab within preoperative physiotherapy programs for patients undergoing coronary artery bypass grafting. A study conducted in Santa Catarina^25^demonstrated that EBPrehab (consisting of at least five days of bodyweight resistance exercises and breathing exercises) combined with Phase I cardiac rehabilitation significantly reduced hospital stays, time to extubation, and the incidence of postoperative complications — including pneumonia, pleural effusion, and atelectasis —compared to Phase I cardiac rehabilitation alone. Additionally, two studies from São Paulo revealed that an EBPrehab program incorporating inspiratory muscle training can shorten postoperative recovery time^30^and improve lung function.^26^

Despite these promising findings, the preoperative cardiac rehabilitation (or “cardiac prehabilitation”) remains underutilized in Brazil.

### Cardiac prehabilitation in Brazil: An emerging need

Despite over four decades of research supporting the benefits of prehabilitation,^2^— including its recommendation in international guidelines for patients undergoing elective cardiac surgery^5 , 6 , 16^— prehabilitation has yet to be recognized as a valuable intervention opportunity in Brazilian guidelines.

The *South American Guideline for Cardiovascular Prevention and Rehabilitation (2014)* ^49^recommends postoperative cardiac rehabilitation for procedures such as coronary artery bypass grafting and cardiac transplantation, yet makes no mention of preoperative interventions like prehabilitation. Similarly, Brazil’s most current comprehensive document on the subject, the *Brazilian Guideline for Cardiovascular Rehabilitation (2020)* ,^50^omits prehabilitation as a recommended practice.

This oversight extends to other key guidelines:

- The *3rd Brazilian Guideline of Cardiac Transplantation (2018)* ^51^focuses solely on medical preoperative evaluation without referencing prehabilitation programs.
- The updated *Guideline for Perioperative Cardiovascular Evaluation (2024) by the Sociedade Brasileira de Cardiologia* ^52^emphasizes risk assessment but fails to include prehabilitation referrals — even for high-risk patients. Notably, its sole mention of physical rehabilitation applies exclusively to frail patients undergoing non-cardiac procedures.

This persistent exclusion in national guidelines represents a critical missed opportunity. Given the growing evidence demonstrating prehabilitation’s efficacy in improving surgical outcomes, Brazil stands to benefit significantly by aligning its clinical practice with international standards through the formal incorporation of prehabilitation protocols.

### Anticipated challenges and proposed solutions for implementation in clinical routine

The implementation of cardiac prehabilitation programs in Brazil will inevitably face challenges. Although specific barriers to cardiac prehabilitation participation are still unknown, the existing literature highlights systemic challenges in the delivery of cardiac rehabilitation services.

Cardiac rehabilitation services in low- and middle-income countries are insufficient to meet the growing cardiovascular disease burden, with low utilization even where available. While barriers exist across all settings, low- and middle-income countries face greater challenges due to resource constraints, weak health systems, and affordability issues.^53^Key implementation barriers include low patient motivation, environmental and dietary challenges, infrastructure gaps, and health literacy disparities. Limited insurance coverage further restricts access and long-term rehabilitation potential.^54 , 55^For elderly frail patients, prehabilitation barriers include transportation limitations, lack of social support, and information overload.^56 , 57^

Given Brazil’s limited cardiac rehabilitation infrastructure for centralized multimodal prehabilitation programs, adaptive implementation strategies are essential. Challenges associated with center-based programs could be mitigated through decentralized models and/or teleprehabilitation, which not only address these barriers but may also enhance cost-effectiveness.^58 , 59^Future research should explore locally adapted models leveraging interprofessional collaboration. For safety considerations, moderate-to-high-risk patients should be referred to a hospital or center-based programs, whereas low-risk patients awaiting elective surgery could receive coordinated, decentralized multidisciplinary care in community settings or via telerehabilitation. Such programs should integrate components from the medical, physiotherapeutic, nutritional, and psychological domains, depending on individual needs, to ensure comprehensive support.

### Future perspectives: Advancing cardiac prehabilitation in Brazil

While gaps in evidence persist and no consensus exists on optimal prehabilitation programs, international medical societies increasingly recognize the urgent need for high-quality studies in this field.^5 , 6^

Recent years have seen numerous multinational initiatives investigating effective prehabilitation strategies for cardiac patients. Notable programs include multimodal approaches (ERAS,^5^PREHAB,^60^PREQUEL,^61^PRECOVERY,^62^PrEPS TRIAL,^63^Heart-ROCQ^64^) and research in teleprehabilitation,^65 , 59^which show promise in improving outcomes for cardiac surgical patients, including those undergoing heart transplantation.^66^

To establish an evidence-based prehabilitation framework tailored to Brazil’s population and healthcare realities, the following priorities should be advanced:

### Key Rrecommendations for Brazil

1. **Guideline Integration**
  - Formal recognition of multimodal prehabilitation in national cardiovascular surgery and cardiac rehabilitation guidelines as a valuable intervention opportunity for elective cardiac surgery.
2. **Population-Specific Research**
  - Investigation of Brazil’s unique epidemiological and cultural factors to ensure prehabilitation programs address local health disparities and resource constraints.
3. **Multidisciplinary Implementation**
  - Establishment of specialized prehabilitation teams (cardiologists, physiotherapists, nutritionists, psychologists) to design contextually adapted programs aligned with Brazil’s healthcare infrastructure.
4. **Pilot Programs in Academic Centers**
  - Deployment of prehabilitation initiatives in university hospitals and cardiac rehabilitation centers to synergize clinical care with research while testing feasibility.
5. **Expansion to Non-Open-Heart Procedures**
  - Rigorous evaluation of prehabilitation’s role in percutaneous coronary interventions, transcatheter aortic valve implantation, and other minimally invasive cardiac procedures.
6. **Exercise Modality Optimization**
  - Comparative studies to determine the safety and efficacy of various training components (aerobic, resistance, and/or inspiratory) within multimodal prehabilitation frameworks.

## Conclusion: A call to action for cardiac prehabilitation in Brazil

Despite growing evidence supporting preoperative rehabilitation for cardiac surgery, Brazil’s healthcare system has not fully implemented this approach. While international guidelines increasingly recognize prehabilitation as a valuable component of surgical preparation,^5 , 6 , 16^its absence in Brazilian cardiovascular guidelines^49 - 52^represents a critical gap in patient care standards. The Brazilian medical community, together with the other health care professionals, stands at a pivotal moment, by:

1. **Formally incorporating** prehabilitation into national cardiac care guidelines.
2. **Development of evidence-based protocols** tailored to local population needs and healthcare infrastructure.
3. **Establishing multidisciplinary programs** in academic health centers.

By embracing these advances, Brazil has the potential to significantly improve cardiac surgical outcomes. The success of international multimodal programs^60 - 64^and emerging teleprehabilitation models^59 , 65 , 66^provides both a roadmap and justification for this evolution. Future research should particularly focus on:

- standardizing outcome measures for Brazilian populations,
- evaluating cost-effectiveness in both public and private healthcare systems and
- exploring applications in minimally invasive cardiac procedures, in order to improve the existing knowledge and optimize the healthcare situation.
